# P-444. Population Pharmacokinetics (PPK) Analysis of Sulbactam-Durlobactam (SUD) to Support Dose Selection for Evaluation in a Clinical Trial in Pediatric Patients with Acinetobacter Baumannii-Calcoaceticus Complex (ABC) Infections

**DOI:** 10.1093/ofid/ofaf695.659

**Published:** 2026-01-11

**Authors:** Kajal B Larson, John O’Donnell, Angela Tanudra, Anthony P Cammarata, Christopher M Rubino

**Affiliations:** Entasis Therapeutics Inc., an affiliate of Innoviva Specialty Therapeutics, Inc., Waltham, MA; AUROBAC Therapeutics, Boston, Massachusetts; Innoviva Specialty Therapeutics, Inc., Waltham, MA; Institute for Clinical Pharmacodynamics, Schenectady, New York; Institute for Clinical Pharmacodynamics, Schenectady, New York

## Abstract

**Background:**

SUD is a bactericidal β-lactam/β-lactamase inhibitor combination approved in the United States for the treatment of hospital-acquired and ventilator-associated bacterial pneumonia caused by ABC in patients aged 18 years and older. No pediatric data on SUD are currently available from a clinical trial. Model-based simulations were performed to identify doses for Study CS2514-2023-002, a Phase 1b clinical trial evaluating the PK, safety, and tolerability of SUD in pediatric patients (NCT06801223).

**Figure:**
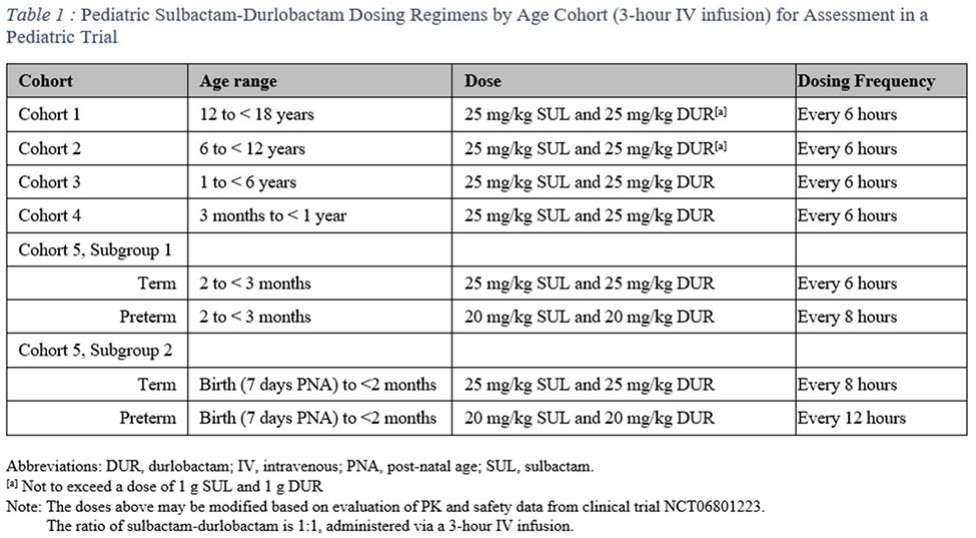

**Methods:**

A PPK model developed using data from eight Phase 1-3 trials in adults was adapted using allometry to predict optimal SUD dosing regimens in pediatric patients from birth (28 weeks of gestation) to < 18 years of age. A dataset of 8000 hypothetical pediatric patients with age- and sex-appropriate body size based on CDC growth charts and normal renal function was created. Renal function was estimated using the Rhodin formula using free fat mass. Model-based simulations were performed to assess the pediatric exposures of SUD that achieved a probability of PK/pharmacodynamic (PD) target attainment (PTA) of ≥ 90% with a similar median exposure as in adults.

**Results:**

Dosing regimens (Table 1) resulted in PTA ≥ 90% and SUD exposures that were predominantly contained within the 5^th^ and 95^th^ percentiles of adult exposures based on peak and systemic exposures on Day 1 and Day 3 (steady state). As pediatric data are collected from the ongoing pediatric clinical trial, the PPK model will be updated to verify that adequate SUD exposures are achieved and to support dose modifications, if warranted.

**Conclusion:**

Model-based simulations were conducted based on allometrically scaling the published adult SUD PPK model, and dosing regimens that are predicted to yield safe and efficacious exposures were derived; these SUD dosing regimens are being evaluated in a Phase 1b clinical trial.

**Disclosures:**

Kajal B. Larson, PhD, Innoviva Specialty Therapeutics, Inc: employee|Innoviva Specialty Therapeutics, Inc: Stocks/Bonds (Public Company) John O'Donnell, BS, AUROBAC Therapeutics: employee|AUROBAC Therapeutics: Ownership Interest|AUROBAC Therapeutics: Stocks/Bonds (Private Company)|Innoviva Specialty Therapeutics, Inc: Stocks/Bonds (Public Company) Angela Tanudra, MS, Innoviva Specialty Therapeutics, Inc: employee|Innoviva Specialty Therapeutics, Inc: Stocks/Bonds (Public Company) Christopher M. Rubino, PharmD, A&G Pharma: Grant/Research Support|AiCuris Anti-infective Cures AG: Grant/Research Support|Albany College of Pharmacy and Health Sciences,: Grant/Research Support|Albany Medical College: Grant/Research Support|AN2 Therapeutics: Grant/Research Support|Antabio SAS: Grant/Research Support|Apogee Biologics, Inc.: Grant/Research Support|Arcutis Biotherapeutics, Inc.: Grant/Research Support|B. Braun Medical, Inc.: Grant/Research Support|Basilea Pharmaceutica: Grant/Research Support|Cumberland Pharmaceuticals, Inc.: Grant/Research Support|Debiopharm: Grant/Research Support|Elion Therapeutics, Inc.: Grant/Research Support|Entasis Therapeutics: Grant/Research Support|Excalibur Pharmaceuticals, Inc.: Grant/Research Support|Fedora Pharmaceuticals: Grant/Research Support|Genentech: Grant/Research Support|Global Antibiotic Research & Development Partnership: Grant/Research Support|Inotrem: Grant/Research Support|Insmed, Inc.: Grant/Research Support|Institute for Clinical Pharmacodynamics, Inc.: Ownership Interest|Invivyd, Inc.: Grant/Research Support|Iterum Therapeutics Limited: Grant/Research Support|Kaizen Bioscience: Grant/Research Support|Lassen Therapeutics, Inc.: Grant/Research Support|Matinas Biopharma: Grant/Research Support|Meiji Seika Pharma Co., Ltd.: Grant/Research Support|Melinta Therapeutics: Grant/Research Support|Nabriva Therapeutics AG: Grant/Research Support|National Institutes of Health: Grant/Research Support|Novobiotic Pharmaceuticals LLC.: Grant/Research Support|Paratek Pharmaceuticals, Inc.: Grant/Research Support|Pfizer, Inc.: Grant/Research Support|Praxis Precision Medicines, Inc.: Grant/Research Support|pRxcision, Inc.: Ownership Interest|PTC Therapeutics: Grant/Research Support|PureTech LYT 100, Inc.: Grant/Research Support|Qpex Biopharma: Grant/Research Support|Renibus Therapeutics: Grant/Research Support|Sagimet Biosciences, Inc.: Grant/Research Support|Schrodinger, Inc.: Grant/Research Support|Sfunga Therapeutics: Grant/Research Support|Shionogi, Inc.: Grant/Research Support|Spero Therapeutics: Grant/Research Support|Spruce Biosciences, Inc.: Grant/Research Support|UCB Biosciences, Inc.: Grant/Research Support|United States Food and Drug Administration: FDA Contract Number: 75F40123C00140|University of Wisconsin: Grant/Research Support|UT Southwestern: Grant/Research Support|VenatoRx Pharmaceuticals, Inc.: Grant/Research Support|Wockhardt Bio AG: Grant/Research Support|Zogenix International: Grant/Research Support

